# Supplementary material for: Investigating the test-retest reliability of Illinois Agility Test for wheelchair users
Source: PLoS One. 2020 Oct 29;15(10):e0241412. doi: 10.1371/journal.pone.0241412 (PMC7595385; doi:10.1371/journal.pone.0241412)
Supplement: Attachment — Submitted filename: Response to Reviewers.docx [file pone.0241412.s002.docx]

Sept. 2, 2020

The Editor

PLOS ONE

Investigating the test-retest reliability of Illinois Agility Test for wheelchair users

Zohreh Salimi and Martin Ferguson-Pell

Dear Dr. Jan,

We would like to thank you for sending us the second letter of review for the above paper on Aug. 17, 2020, and also for giving us the opportunity of sending a revised version of our manuscript. We also would like to thank the reviewers for the thorough reviews and the very helpful and constructive comments.

We have applied the reviewers’ comments and addressed them in the below table. The revised manuscript is uploaded alongside this letter (one with changes tracked and one with all revisions applied and accepted). I hope that the revised version is acceptable for publication in PLOS ONE.

Please do not hesitate to contact me if I can provide any further information about this study.

Sincerely yours,

Zohreh Salimi

|  | Comments | Responses |
| --- | --- | --- |
| Reviewer #1: |  |  |
|  | *Introduction:* |  |
| 1 | Lines 94-96: Minor wording. Consider moving “wheelchair maneuvering” before “training athlete wheelchair users” as maneuvering is the primary focus of the introduction and manuscript as a whole. | Done. |
|  | *Methods:* |  |
| 2 | Lines 115 and 116: Using “average” in demographics is unnecessary after the authors indicate data listed are mean ± SD. | Very right. Corrected. |
| 3 | Line 127: Consider using mean ± SD for consistency. | Sure. Corrected. |
| 4 | Justification is needed for why participants performed an agility test at a comfortable speed (rather than done as quickly as possible [Line 134]) | Since completion time is not a great concern for wheelchair users when accomplishing wheelchair manoeuvres. Rather, other factors like how well they can finish the task without overusing their shoulders, are of more concern to them.  An explanation is added to the manuscript [lines 141-142]. |
|  | *Results:* |  |
| 5 | The ICCs shows good to excellent reliability, which is the main outcome of the study. If ICCs are high for most variables, this should be the sole justification for reliability. Concerns remain for the interpretation of MANOVA tests. Why supplement statistical testing with MANOVAs that fail to reject the null hypothesis? This would also eliminate any verbiage needed regarding Bonferroni corrections. | As the reviewer pointed out, the main evidence we are presenting to show the similarity here is the *ICC* between 16 variables, which is a standard statistical approach for proving reliability. The MANOVA analyses, however, have been used to only confirm the proven idea by adding extra supporting material. Fortunately, the MANOVA results for *ALL 37 variables were favorable and backed the main hypothesis of the study.*  From the MANOVA results, we concluded that the parameters were not different. We need to emphasize that “not rejecting the null hypothesis” was favorable for our study. This should clear up the misunderstanding and show that using MANOVAs not only did not weaken but also strengthen the results of the study. |
|  | *Discussion:* |  |
| 6 | Lines 380-386: References are needed for these statements. | 6 references were added. |
| 7 | Future research is likely needed with non-able-bodied wheelchair users (i.e., participants with disabilities) to ensure repeatability of the IAT, especially if authors suggest that IAT can be used for future wheelchair maneuvering research. | A paragraph is added regarding this issue on lines 371 to 376. |
| Reviewer #2: |  |  |
| 8 | I have no problems with the statistical procedures as they are presented in the revision, with one very minor exception. By my calculations, the power analysis conducted on page 5 suggests a sample size of 11.69 to achieve power of 0.8, so technically you should have used 12 subjects to achieve 80% power, not 11. As I said, though, I consider this a minor point. | You are right. Rounding it to 12 was a better practice. For greater clarity, I denoted in the manuscript that the exact result of the formula was 11.63 (according to this:  1.5+((1.64+.84)/((ln((1+.6)/(1-.6))/2)-(ln((1+.9)/(1-.9))/2)))^2=11.63). |
| 9 | A few grammatical errors remain in the manuscript. | We implemented another round of grammatical check and also applied the grammatical points raised by the first reviewer. |
| Editor: |  |  |
| 10 | Upload your figure files to the Preflight Analysis and Conversion Engine (PACE) digital diagnostic tool | Done. |
